# The Application of Electroencephalogram in Driving Safety: Current Status and Future Prospects

**DOI:** 10.3389/fpsyg.2022.919695

**Published:** 2022-07-22

**Authors:** Yong Peng, Qian Xu, Shuxiang Lin, Xinghua Wang, Guoliang Xiang, Shufang Huang, Honghao Zhang, Chaojie Fan

**Affiliations:** ^1^Key Laboratory of Traffic Safety on Track of Ministry of Education, School of Traffic & Transportation Engineering, Central South University, Changsha, China; ^2^School of Business and Trade, Hunan Industry Polytechnic, Changsha, China; ^3^School of Mechanical Engineering, Shandong University, Jinan, China

**Keywords:** electroencephalogram, distraction driving, emotion driving, fatigue driving, traffic safety

## Abstract

The driver is one of the most important factors in the safety of the transportation system. The driver’s perceptual characteristics are closely related to driving behavior, while electroencephalogram (EEG) as the gold standard for evaluating human perception is non-deceptive. It is essential to study driving characteristics by analyzing the driver’s brain activity pattern, effectively acquiring driver perceptual characteristics, creating a direct connection between the driver’s brain and external devices, and realizing information interchange. This paper first introduces the theories related to EEG, then reviews the applications of EEG in scenarios such as fatigue driving, distracted driving, and emotional driving. The limitations of existing research have been identified and the prospect of EEG application in future brain-computer interface automotive assisted driving systems have been proposed. This review provides guidance for researchers to use EEG to improve driving safety. It also offers valuable suggestions for future research.

## Introduction

It’s well-known that drivers play a crucial role in the driving process, which requires substantial cognitive effort and attention (Lemercier and Cellier, 2008) from the operator’s brain (Chisholm et al., 2008). According to the Statistics of the World Health Organization (WHO), 1.35 million people die from road traffic accidents every year (Tan et al., 2021; Wang et al., 2021).

Fatigue and distraction (Stutts et al., 2001) are thought to be important factors in traffic accidents (Carney et al., 2015). Although the ratio of the accidents caused by fatigue driving varies from about 1% to about 20% in different regions (Pei et al., 2013; Anund et al., 2016), the consequences of traffic accidents caused by fatigue are comparatively more serious, and fatigue driving accounts for a higher proportion in fatal accidents (Armstrong et al., 2010). This is because the fatigue is more likely to be ignored by drivers than other factors (Vanlaar et al., 2008; Miller et al., 2020), which was interpreted as an optimistic bias in some research (Meng et al., 2015). This optimistic bias will make drivers less inclined to rest when they feel tired, and more likely to continue to drive, which increases the possibility of accidents. The data had shown that lane departure crashes caused by driving distraction in the United States account for 20% of officially reported crashes, and accounts for 41% of traffic accident mortality (Fagerberg, 2004). Klauer et al. (2006) found that nearly 80% of collisions and 65% of critical collisions were related to distraction by analyzing the raw driving data of 100 vehicles (). This is because the distraction can slow down the reaction time (RT) of the driver by up to two seconds, thereby raising the risk for accidents significantly (Zwahlen et al., 1988). For instance, the visual distraction can reduce driver’s lateral control ability and the time of looking at the road (Carsten, 2006).

As the driver’s psychological feedback to the traffic environment, emotion is also considered to be the main factor causing traffic accidents. For instance, the congested traffic situation often stimulates the anger emotion generation of drivers, and further induces drivers’ cognitive deficiency (e.g., attentional bias) (Mesken et al., 2007). Anger provocation leads to an increased tendency to underestimate the potential traffic hazards (Stephens and Groeger, 2009). With the diversification of modern life style, emotional changes are more prominent, including not only negative emotions, but also extreme emotions, which will lead drivers to an abnormal driving level. As is evident from the quoted accident statistics, the status of drivers is the most commonly contributing factor in fatal accidents worldwide, moreover, an ineffective driving status (e.g., fatigue, distraction, anger) plays an important role in the sequence of events leading to many of the accidents.

Poor driving status (Abdoli et al., 2015) (e.g., distracted and drowsy) may have a significant impact on the quality of maneuvers performed, with potentially catastrophic consequences for both the passenger and the driver. Roughly speaking, the two tasks may have similar visuomotor and cognitive characteristics that may cause similar adjustments in workload (Hancock and Verwey, 1997) profiles, affecting driver performance (Horberry et al., 2006), which may lead to potentially catastrophic consequences. Situations exist that require alertness (Smith et al., 2000) from the driver for noticing issues as well as accurate judgment for tackling them. Hence, the need for a continuously improved understanding of driver behavior and how to optimize driving performance is particularly important. Therefore, effective monitoring and regulation of drivers’ bad driving state and in-depth revelation of the nature of dangerous driving behavior have become a research hotspot in the field of road traffic safety.

**FIGURE 1:**
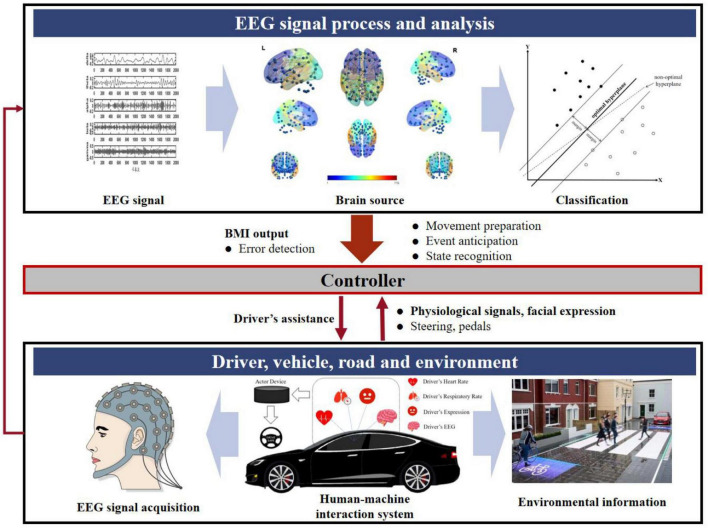
Driving assistance system considering driving states.

The most common methods for driver status detection can be divided into the vehicle-based, video-based, and physiological signals-based techniques. The self-assessment (Eby et al., 2003) of fatigue, driving physical features (Ji et al., 2004; Peng et al., 2022b), facial features (Lee and Chung, 2012), voice intonation features (Krajewski and Nöth, 2007), and neurophysiological features (Fan et al., 2021) measures may contain camouflaged data in the research process, leading to certain unreliability of research conclusions. Among these methods, the neurophysiological measurements and their associated features are the most effective, which have been widely used in attempts to describe different human mental statuses and to estimate the activity of central nervous systems related to driving performance. The physiological signals include electroencephalography (EEG), electrocardiography (ECG), electromyography (EMG), electro-oculogram (EOG), Phonocardiogram (PCG), galvanic skin response (GSR), respiration rate (RT), and skin temperature (ST), all of these physiological data (Shen et al., 2008) that have been widely used in attempts to identify and detect human statuses (Jap et al., 2009; Gunes et al., 2011; Cheng et al., 2022b; Fu et al., 2022; Peng et al., 2022a). Among these methods, the EEG signals represent the most promising way to detect driver states since they can reflect the physiological activity of the human brain more intuitively and are more accurate due to strong immunity to artifacts (Chavarriaga et al., 2018). EEG is an electrophysiological signal, and different mental activities, emotions or external activities can affect the changes of brain waves. Compared with other physiological signals, EEG signals can reflect the physiological activity of the human brain more intuitively, which are called the “gold standard” for evaluating human cognitive state due to their advantages of high temporal resolution, non-invasiveness, low-cost properties (Xing et al., 2020). Moreover, EEG uses a simple and subject-acceptable method to obtain data that can be used for driver state perception analysis. Therefore, EEG signals have become a common focus for future intelligent transportation-assisted driving and brain-computer interface fields (Cheng et al., 2022a; Fan et al., 2022). An optimal human-machine symbiotic interaction, where the vehicle can consider the driver’s goals and preferences, can be achieved by fully exploring the intrinsic correlation between driving states and physiological/psychological signals. The block diagram of the driving assistance system considering driving states is shown in Figure 1. With full consideration of the environmental information, driver’s physiological signals (e.g., EEG, heart rate, respiratory rate) and facial expressions, and the driving states inferred by the recognition modules, especially a brain-machine interfaces (BMIs), the controller of driving assistance system can determine the type and level of assistance it provides.

This study systematically reviews these EEG-based researches of adverse driving states (e.g., fatigue, distraction and emotion) from the theoretical, technical and applied levels, including their definitions, causes, effects on driving behavior, generation mechanism from the EEG level, detection methods based on EEG, etc. Furthermore, the key EEG theoretical concept is also introduced. This study can help road traffic safety researchers to understand the nature of bad driving behavior, grasp the development and dilemma of adverse driving state detection in the field of brain science, and determine the future development perspectives. The remainder of the paper is organized as follows: Section “Basic Theory of Electroencephalography” introduces the basic theory of EEG, sections “Study of Fatigue Driving Based on EEG,” “Study of Distraction Driving Based on EEG,” and “EEG Based Studies in Emotional Driving” respectively introduce the main applications of EEG in the traffic field such as fatigue driving, distracted driving, and emotional driving; section “Discussion, Conclusion, and Future Prospects” summarizes the development trend and limitation of adopting EEG for drivers in the future transportation field.

## Basic Theory of Electroencephalography

To begin with, we introduce the theories of EEG signals, the types of EEG electrodes, brain waves or neural oscillation. EEG is a method of recording brain activity using a net of regularly spaced electrodes and is the *superimposed of* postsynaptic potentials of many neurons in the cerebral cortex (Shan et al., 2018; Hou et al., 2021; Liu et al., 2022).

**FIGURE 2:**
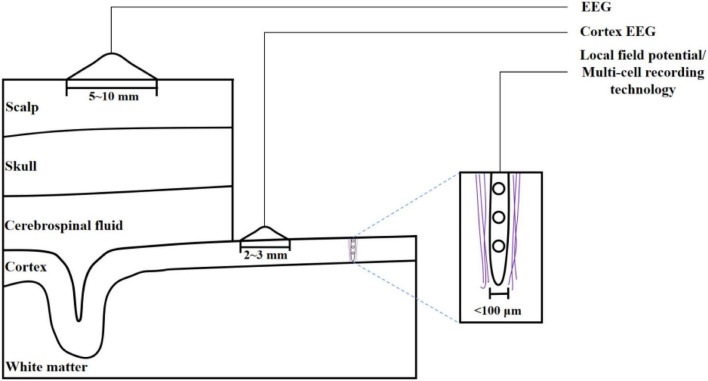
Three brain activity recording techniques.

**FIGURE 3:**
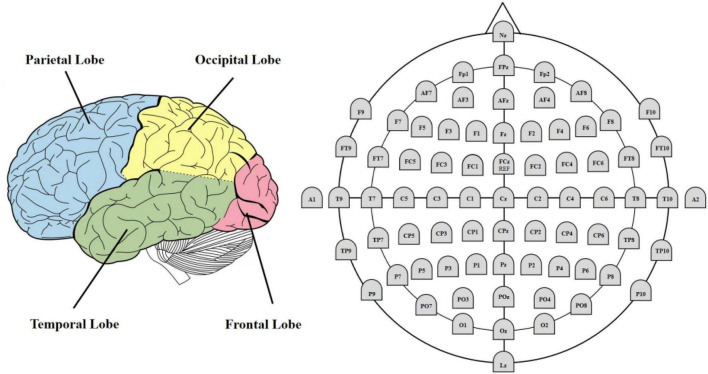
EEG 10-20 international system.

Electrocorticogram (ECoG) refers to similar records obtained directly from the cerebral cortex or dura mater. Local field potential (LFP) refers to the insertion of a small electrode into the brain to record electrical signals generated by brain activity. All three techniques record postsynaptic potentials generated by neuronal activity (Buzsáki et al., 2012). Figure 2 shows the differences in three different types of brain activity recording techniques. This paper mainly discusses EEG, which is the most widely used in neuroscience research (Henry, 2006). Figure 3 shows the EEG 10–20 international system, which is widely used to regulate the position of electrodes (Acharya et al., 2016). The system specifies the standardized position of 75 electrodes on the scalp, each at 10 and 20% points along the longitude and latitude lines, respectively. The name of the electrode consists of two parts. The English letter is the approximate area corresponding to the electrode: frontal pole (Fp), frontal lobe(F), central region(C), parietal lobe(P), occipital lobe(O), temporal lobe(T), and the ending number represents the distance to the midline. The higher the number, the farther away from the midline. Odd numbers are used in the left hemisphere and even numbers are used in the right hemisphere (the division between left and right is based on the subjects’ perspective). According to its spatial location, the cerebral cortex is divided into frontal lobe, parietal lobe, temporal lobe and occipital lobe.

**FIGURE 4:**
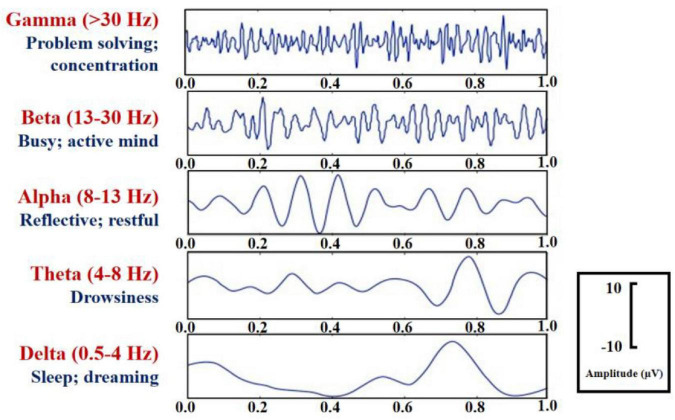
EEG wave band categories.

Analysis of brainwaves and their decomposition in different frequency bands are often used to assess changes in the “*intrinsic dynamics*” of the subject while performing simple cognitive or sensor-motor tasks (Kim et al., 2020). EEG is the *gold standard* for brain activity measurement and is considered a good indicator of mental status (Shalash, 2019). The amplitude or power of brain waves in specific brain regions, as well as in different frequency bands throughout the brain, has been shown to be reliably associated with different cognitive processes (Züst et al., 2019). Brain waves are usually divided into *five* different waves according to their frequencies, namely the Gamma (30–42 Hz), Beta (13–30 Hz), Alpha (8–13 Hz), Theta (4–8 Hz) and Delta (0.5–4 Hz) waves (Holm et al., 2009) as shown in Figure 4, of which Delta and Theta waves are called slow waves, which usually appear when a person is asleep or in meditation. The alpha wave is the basic rhythm of normal brain waves, which occurs when the brain is awake and relaxed. The beta wave is a fast wave, which usually occurs when a person is mentally stressed or hyperactive. It has been shown that a decrease in alertness and a worse performance are associated with increased EEG power spectra in the theta band and changes in EEG alpha power (Gale et al., 1977). In addition, Okogbaa et al. (1994) noted that increased EEG power spectra in the beta band were associated with increased alertness and arousal, alpha waves occurred during relaxation conditions with reduced levels of attention in a drowsy but awake state; and theta waves occurred primarily in the sleep state. Many studies on EEG studies have also been conducted by extracting the characteristic quantities of these EEG waves data analysis.

Currently, there are numerous applications of EEG research results in the study of traffic driving behavior, mainly focusing on traffic safety. The driver is an important component of the traffic system, and in the field of intelligent transportation, vehicle driver assistance is a key component of the traffic system, and the development of its vehicle driver assistance systems has mostly focused on monitoring the driver’s driving state (Namazi et al., 2019). In the field of traffic flow theory, some exploratory studies have been conducted to incorporate the driver’s physiological and psychological perceptions during dynamic driving as a parameter in traffic flow models (Tang et al., 2012). EEG signals can visually reflect the physiological activity of the brain, which further reflects the psychological perceptual activity of a person. Therefore, applying the results of EEG signal research to the field of traffic driving behavior can effectively improve driver cognition and contribute to the development of relevant traffic models that incorporate driver perception. Next, we discuss the research on driver brain waves under fatigue driving, distracted driving, and emotional driving, respectively.

## Study of Fatigue Driving Based on EEG

Whether the driver’s fatigue state can be accurately detected is very important to ensure driving safety. Studies have shown that driving fatigue may be caused by driver mental overload (Hu and Lodewijks, 2021) or mental underload (Ahlstrom et al., 2021). Mental overload usually occurs because of continuous, long-time concentrated work. And although the effect of mental underload on fatigue is not as obvious as mental overload, it will still aggravate driver’s drowsiness in situations such as night driving or driving straight for a long time (Young and Stanton, 2007; Solls-Marcos et al., 2017). Some researchers stated that mental underload would impair the driver’s ability to distribute attention resulting in an inevitable collision (Nilsson, 1996; Young and Stanton, 2002). Despite there being some differences between fatigue, drowsiness and sleepiness, they usually mean the same thing in EEG-based research. In this section, we will discuss the development history and application of EEG fatigue detection.

At first, studies were mostly aimed at finding a qualitative relationship between fatigue and EEG signals. Lal and Craig (2001) analyzed and obtained the changing characteristics of EEG in different fatigue levels based on the average awake-stage EEG activity, and found that the activity of delta wave and theta wave increased in the fatigue stage. Shen et al. (2008) detected the drivers’ mental state *via* EEG and proved that using EEG to estimate the drivers’ fatigue level is feasible. Yeo et al. (2009) carried out a driving simulating experiment which indicated that there was an alpha loss phenomenon when the subjects were sleepy.

Later, research were performed to discover possible EEG-based fatigue parameters or features and to quantitatively analyze the link between fatigue and EEG signal. Jap et al. (2009) proposed four kinds of algorithms (i) (θ + α)/β, (ii) α/β, (iii) (θ + α)/(α + β), and (iv) θ/β to detect the fatigue, and found that algorithm revealed a larger increase in all four algorithms when fatigue was detected. They also examined the possibility of using features of alpha, beta, delta and theta wave to identify fatigue, and proposed four kinds of combination indices θ/α, β/(α + β), (β + α)/β and (θ + α)/(α + β). Combining these indices with driving time, the results showed that the change of θ/(α + β), β wave and θ wave could be applied to detect fatigue together (Jap et al., 2011). These studies brought forward new indices by combining EEG signal with its physiological characteristics and verified the effectiveness of these indices subsequently. It is an efficient way to find new fatigue indicators for later studies.

Further, the time-dependent EEG signal is transformed into a spectrum of EEG power varying with frequency, so as to directly analyze the changes of different frequencies or frequency bands related to specific brain activity is called frequency domain analysis. In the field of driving safety, EEG is usually divided into delta rhythm (0.5–4 Hz), theta rhythm (4–8 Hz), alpha rhythm (8–13 Hz), beta rhythm (13–30 Hz), and gamma rhythm (above 30 Hz) according to the length of the period or the frequency. The EEG study can be analyzed by directly extracting these rhythms or the associated signal characteristic quantities made by the combination of these rhythms. Ahlström et al. (2018) found by power spectral density estimation that in the same road environment, due to increased subjective sleepiness in night-time driving, EEG alpha rhythm content increased in the same road environment. In addition, it is becoming a common analysis method to focus on the spatial distribution of EEG signals on the scalp surface by combining the frequency domain information and extracting the corresponding feature indicators to analyze the activity status of different brain regions. In addition, combining frequency domain information with spatial information to focus on the spatial distribution of EEG signals on the scalp surface and extracting corresponding characteristic indicators to analyze the activity status of different brain regions is also becoming a common analysis method. Charbonnier et al. (2016) used the spectral information of EEG signals obtained in six brain regions to calculate the spatial covariance matrix of EEG in different brain regions over 20 s and transformed it into a driving fatigue index varying between 0 and 1. The conclusion showed that the alpha rhythm-based driving fatigue index can be used as a characteristic indicator for an accurate assessment of mental fatigue over a long period of time.

There are also studies that extract novel features. For example, a new feature ξ 20 is generated by bispectrum analysis of the 30 s time window (Vinayak et al., 2010). This feature can track the gradual development of drowsiness until standard sleep stage I. Another new feature, IEBW, is generated by PTFD analysis and has a 10-s time window (Yoshida et al., 2007). This feature can distinguish between the awake state and the sleepy state and the normal sleeping state. However, these two features require intensive computing, so their real-time performance needs to be further evaluated.

Based on these features and parameters, researchers had proposed methods of driver state classification. Vuckovic et al. (2002) Used the complete spectral density of EEG as the input of artificial neural network to automatically identify the driver’s alert state and sleepy state. Guo et al. (2016) argued that α/β had the highest correlation with reaction time, and used a Gray correlation to enhance the accuracy of fatigue classification, finally achieving an accuracy of 86%. However, there were only two EEG experts to observe the EEG waveform to subjectively judge the subjects’ awake and sleepy states, which is a lack of objectivity. Mu et al. (2017) estimated the feasibility of an entropy-based feature extraction method and acquired an accuracy of 98.75% using Support Vector Machine(SVM) classification algorithm. Although SVM performs well in generating decision surfaces when processing high-quality data, it isn’t suitable for complex invariance. San et al. (2016) proposed a hybrid deep genetic model to remedy for the deficiency of SVM in processing complex invariance. Chen et al. (2018) combined synchronization likelihood with minimum spanning tree, and employ them in feature recognition and classification. Zeng et al. (2019) proposed a new detecting method of driver state based on LightGBM algorithms and gained a better performance in classification and decision efficiency compared to SVM, convolutional neural network (CNN) and other traditional classification methods. More and more studies also prefer to use time-frequency analysis to obtain information of EEG, where wavelet analysis and wavelet packet analysis become the focus of attention of various researchers. B and Chinara (2021) extracted time-frequency features from EEG signals in the selected channel using wavelet packet transform, and finally proposed a drowsiness detection model based on single-channel EEG signals. Wang proposed a driving fatigue detection method based on multi-non-linear feature fusion strategy to evaluate the degree of driver fatigue (Wang et al., 2020). These models not only explore new feature indexes, but also reduce the dimensionality of EEG data, greatly improve the running speed of the model, and are of practical significance.

On the basis of the improved discrimination and prediction methods of driver fatigue, the research of brain fatigue monitoring system based on EEG began to emerge. Lin et al. (2005) established a system based on EEG power spectrum analysis, independent component analysis and fuzzy neural network model. The system could evaluate the driver’s cognitive state and predict the driver’s driving behavior at the same time. Lin et al. (2011b) optimized the process of artifact removal and brain source selection. On this basis, the driver fatigue recognition model is established by using independent component analysis and self-organizing map, and the accuracy is about 90%. Raut and Kulkarni (2014) designed a brain computer interface system that can detect driving fatigue in real time, which can monitor the driver’s sleepiness and send an alarm to the driver when it is found, so as to prevent traffic accidents (Raut and Kulkarni, 2014). Some real-time and practical driver fatigue identification devices for car drivers, train drivers and pilots were also designed. He et al. (2014) developed a real-time fatigue monitoring device based on sedentary EEG, which can run under Android system. Zhang et al. (2017) designed a portable EEG-based fatigue detection device for high speed train drivers. The device would collect drivers’ EEG and send the raw EEG data to the computer where the data is processed to detect drive fatigue. Once fatigue was detected, the device would send a message to wake the driver up. Some studies integrated EEG with other human features in order to obtain higher recognition accuracy and processing speed. Fu et al. (2016) combined EEG with Electromyogram(EMG) and respiratory signal, which significantly increase the posterior probability.

However, for the sake of safety, most driving fatigue simulation experiments were carried out under laboratory simulation conditions. And researchers had built driver fatigue detection systems based on EEG in the laboratory environment. Although the development process of drowsiness is similar to the real on-road environment (Fors et al., 2018), some researchers suggested that subjects tended to have higher subjective and physiological drowsiness level using a driving simulator (Hallvig et al., 2013). As a result, in order to ensure security, more factors must be involved in the migration from laboratory environment to real environment. In addition, for the real environment, the probability of identification error should be reduced as much as possible. Hybrid measurement based on EEG was considered to provide a more reliable solution (Dong et al., 2011), and hybrid measurement reduced the number of identification errors, thus improving the availability of the system.

Timeliness is a major challenge for driver fatigue detection. In order to solve this problem, it is necessary to use not only a shorter data processing time window, but also an intelligent data mining model to ensure that the driver’s drowsiness is estimated in time (Wei et al., 2015). From the point of view of timeliness, EEG is more suitable for driver fatigue detection applications. The physiological reason behind the short time window of EEG analysis is its direct relationship with drowsiness. The key is that for EEG itself, the time window of feature extraction is directly related to the timeliness of the fatigue detection system.

In addition, the latest developments in EEG dry sensors, low-power integrated circuits and wireless communication technologies have moved EEG-based fatigue detection from research to practical applications. Chen et al. (2017) proposed a new EEG method based on main band power spectral density (PSD) to estimate the mental load of tasks, which reliably evaluates the cognitive needs of construction tasks. With the development of wireless and wearable EEG devices, we believe that EEG-based fatigue detection system is a more promising research field under natural driving conditions.

Electroencephalogram related technologies make it possible to develop driver fatigue detection systems with higher precision and lower time delay. Due to the non-hidden characteristics of EEG, driver fatigue can be identified or predicted before external performance, which effectively reduces the time of detection-feedback loop, gives drivers more reaction time and reduces the incidence of accidents. In the long transition period between manual driving and automatic driving, the detection and feedback of driver fatigue based on EEG can effectively reduce the incidence and mortality of traffic accidents. The experimental environment and corresponding research methods of some studies are summarized and sorted out as shown in Table 1.

**TABLE 1:** Research summary of fatigue driving based on EEG.

Author (year)	Objective	Environment	Participants	EEG signal analysis method	Data analysis method
Lal and Craig, 2001	Relationship between EEG and fatigue	Static test	35	Power spectrum analysis	ANOVA; *Post hoc* analysis
Vuckovic et al., 2002	Discrimination and classification of fatigue levels	Static test	17	manual judgment	T-test
Lin et al., 2005	Driving fatigue monitoring system based on EEG	Driving simulation	10	Power spectrum analysis; Independent component analysis	Correlation analysis
Shen et al., 2008	Fatigue measurement	Static test	10	FFT; Power spectrum analysis	T-test
Yeo et al., 2009	Driver fatigue classification	Driving simulation	20	FFT; Power spectrum analysis	–
Jap et al., 2009	EEG fatigue parameters	Driving simulation	52	FFT	ANOVA
Kar et al., 2010	EEG fatigue parameters	Real on-road experiment&Driving simulation	40	Wavelet transform; Entropy based analysis	Average deviation; Standard deviation
Jap et al., 2011	EEG fatigue parameters	Driving simulation	50	FFT	ANOVA; LSD multi-comparison
Raut and Kulkarni, 2014	Driving fatigue monitoring system based on EEG	–	–	–	–
Wang et al., 2014	Fatigue measurement	Driving simulation	14	Independent component analysis; Self-organizing map	–
He et al., 2014	Driving fatigue monitoring system based on EEG	Driving simulation	18	manual judgment	–
Guo et al., 2016	Relationship between EEG, fatigue and reaction ability	Driving simulation	20	Power spectrum analysis	Cross validation
San et al., 2016	Fatigue measurement	Driving simulation	5	Power spectrum analysis	–
Mu et al., 2017	Fatigue measurement	Driving simulation	12	Entropy based analysis	T-test
Zhang et al., 2017	Driving fatigue monitoring system based on EEG	Driving simulation	20	Wavelet packet transform	–
Chen et al., 2018	Fatigue measurement	Driving simulation	15	Wavelet packet transform	T-test
Zeng et al., 2019	Fatigue measurement	Driving simulation	10	Independent component analysis	Cross validation

## Study of Distraction Driving Based on EEG

The International Organization for Standardization defined distracted driving (Pettitt et al., 2009) as “focusing on activities unrelated to driving, which seriously affects driving behavior.” Lee et al. (2008) defined driving distraction as “a kind of dangerous behavior that drivers turn their attention to activities unrelated to driving tasks, resulting in the decline of drivers’ vision, cognition, decision-making, and operation ability.”

The distraction task may be derived from the external environment (Regan et al., 2008), which is a significant stimulus for bottom-up attention grabbing (Miller and Buschman, 2013). On the other hand, distraction can also be internal, a phenomenon known as cognitive distraction (Chun et al., 2011). However, the driver’s attention capacity is limited, due to this feature, the driver needs to choose the focus of attention, either toward driving or toward distraction. Investigating the origins of distraction and its influence on driving behavior is very important for improving safety on the road. For instance, research in this field could contribute to the development and improvement of advanced driver assistant systems (ADAS) that help to reduce the number of accidents by flexibly adjusting to the current driver state. The National Highway Safety Administration divided driving distraction into visual distraction, auditor*y* distraction, cognitive distraction and physical distraction (Ranney et al., 2001). In the complex and diverse traffic environment, the types of stimuli that cause driver distraction include visual stimulus (Karthaus et al., 2018), auditory stimulus (Caird et al., 2018) and so on. The study found that, compared with auditory stimuli, visual resource conflict is the largest (Wickens, 2002). Various studies have confirmed that visual stimulation has a greater distracting effect (Sodnik et al., 2008). Therefore Human Machine Interface and The Safety of Traffic in Europe had done a lot of research (Carsten, 2006) con the evaluation methods and indicators of drivers’ visual distraction and cognitive distraction. It was found that visual distraction could reduce the lateral control ability and the time of looking at the road; Cognitive distraction would reduce the driver’s steering ability, and improve the driver’s frequency of looking at the center of the road and lane line keeping ability. Previous research (Wali et al., 2013a) showed that researchers had been able to use machine vision to detect and identify visual distraction efficiently and accurately. Compared to visual distraction, the detection accuracy of cognitive distraction is higher since the relevant data is more simple to process (Sonnleitner et al., 2014). Furthermore, it is more easily implemented to monitor driver’s cognitive distraction in real time. For these reasons, EEG-based distraction detection experiment is largely regarded to cognitive detection. And researches on EEG-based distraction detection were mainly about finding the relationship between EEG signal and visual distraction or cognitive distraction.

Due to different types of interference, the research on distracted driving is more detailed than fatigue driving (Young and Salmon, 2012). It includes not only the relationship between EEG and distracted driving and the classification of distracted driving, but also the active site of different interference tasks in the brain and the prediction of the starting and ending time of distracted driving by EEG. At present, research regarding cognitive distraction accounts for the largest proportion, and the experiment on distraction detection is also mainly under driving simulation environment. Moreover, the data processing mainly depends on the theory of probability and statistics. At the same time, because setting different cognitive interference tasks is needed in distracted driving experiments, the EEG activities related to interference events have become the hot spot of distracted driving research.

Electroencephalogramsignals can be extracted from a variety of feature indicators, and the selection of appropriate feature indicators is very important for the content of the study. The Event-Related Spectral Perturbations (EPSP) analysis (Lin et al., 2008) has been widely conducted. Lin et al. (2008) designed random vehicle trajectory offset interference and mathematical calculation interference experiments, and found that when the driver is distracted, there would be an increase of frontal theta and beta activities. Then, further study showed that the increase of frontal theta wave power could be an indicator of the severity of interference during real driving (Lin et al., 2011a). Almahasneh et al. (2014) analyzed the hemispheric data and stated that the right frontal cortex was the most affected area during distracted driving. Therefore, the activation of the right frontal cortex might be regarded as a feasible spatial index that indicates the driver distraction. They later discovered that when the driver was distracted, the frontal lobe electrode pairs and the posterior parts of the brain showed a higher degree of coherency (Almahasneh et al., 2016). Savage et al. (2020) argued that distraction could cause an overall reduction of theta wave activity in occipital part. Wali et al. (2013b) extracted the power spectral density and spectral center of gravity frequency of different wavelets (DB4, db8, Sym8, and coif5). Mean and standard deviation were calculated and an analysis of variance (ANOVA) was performed. The result showed that these two features of Sym8 are highly distinguishable from distraction levels. Using the power spectral density features extracted by Sym8 wavelet, the best average accuracy obtained by subtraction fuzzy classifier was 79.21%. Liu et al. (2016) used the semi-supervised machine learning method to improve the G-mean by 0.0245 compared with the traditional supervised learning method without adding labeled data.

The potentials can be measured by electrodes attached to the subject’s scalp when they are exposed to external visual or auditory stimuli. Such evoked potentials are often referred to as event-related potentials (ERPs) (Donchin, 1979). In studies of driver distraction, ERP is commonly used to measure the amplitude and latency of one or several components of the EEG signal. For example, one study used negative slow waves (NSW), the most negative event-related potentials in the 430-995 ms range on electrodes Fz and Cz, to assess the allocation of neural resources in single- and dual-task conditions (Chan and Singhal, 2015). It was found that the NSW wave amplitude was reduced in the dual-task condition compared to the single-task condition and indicated that the driver shifted cognitive resources from the primary driving task to processing distracting stimuli. In another study comparing P300 wave amplitude with driving difficulty, an increase in difficulty was found to be associated with a decrease in P300 wave amplitude (Chan et al., 2016). Liang and Lin (2018) showed in a study of driver perception of road hazards that there were differences in event-related potentials between hazardous and non-hazardous stimuli in the Pz, Cz, and C3 channels, and in particular, significant differences between hazardous and safe drivers within a time window of 80–100 ms after stimulation. Studies using ERP signals to investigate the association between hazard perception and driver behavior deserve further attention.

Furthermore, the event-related potential P300 (Soltani and Knight, 2000) reflects physiological and psychological functions related to cognitive processes such as perception and memory, and can be divided into two subgroups, P3a and P3b. It is found that when drivers deal with simple interference tasks, there is no significant change in driving behavior, but the amplitude of ERP in EEG signal is significantly weakened, which suggests that stimulus context as defined by the target/standard discrimination difficulty rather than stimulus novelty determines P3a generation. In the driving context, for example, inhibition deficits associated with declines in the cognitive processing of distraction stimuli are reflected in a smaller P3b amplitude (Karthaus et al., 2018). Analyzing the types of event-related synchronization and desynchronization (ERS/ERD) of drivers under auditory interference can provide a new idea for the cognitive model modeling of brain-computer interaction.

Some researchers held the opinion that driving distraction was generally the interaction of two or more types of distractions. Yusoff et al. (2017) discussed the feasibility of a hybrid detection methods using four kinds of common measurement method (driving performance measurement, driver physical measurement, driver biological measurement and subjective reports), and proposed a hybrid measurement method of physical measurement and physiological measurement. They verified that this hybrid method had higher accuracy than other methods in detecting distraction (Yusoff et al., 2017).

Different from other detection methods that rely on external features, EEG-based detection can effectively identify all types of distraction, and has advantages in the detection of cognitive distraction. This feature may enable using a single EEG device to recognize mixed distractions, reduce the number of detection devices, and improve portability. The experimental environment and corresponding research methods of some studies are summarized and sorted out as shown in Table 2.

**TABLE 2:** Research summary of distracted driving based on EEG.

Author (year)	Objective	Environment	Participants	EEG signal analysis method	Data analysis method
Berti, 2010	Relationship between REPs and distraction	Static test	12	Amplitude analysis	T- test; ANOVA
Lin et al., 2008	Relationship between EEG and distraction	Driving simulation	11	ICA; FFT; ERSP	ANOVA
Lin et al., 2011a	Relationship between EEG and distraction	Driving simulation	15	ICA; FFT; ERSP	ANOVA
Lin et al., 2011b	Relationship between EEG and distraction	Driving simulation	16	ICA; FFT; ERSP	ANOVA
Wali et al., 2013b	Relationship between EEG and distraction	Driving simulation	50	Wavelet packet transforms; FFT	Mean ± SD; ANOVA
Almahasneh et al., 2014	The active position of the brain when distracted	Driving simulation	42	Amplitude analysis; Linear discriminant analysis	Paired sample t-test
Liu et al., 2016	Distraction measurement	Real on-road experiment	41	–	–
Savage et al., 2020	Relationship between EEG and distraction	Driving simulation	17	ICA	T-test

## EEG Based Studies in Emotional Driving

Emotional driving is when a driver’s emotional state deviates from the norm driving behavior (Dula and Geller, 2003). Some studies have pointed out that emotion has become one of the main causes of traffic accidents, among which the identification of emotion is the focus of driving emotion research (Villanueva et al., 2015).

**FIGURE 5:**
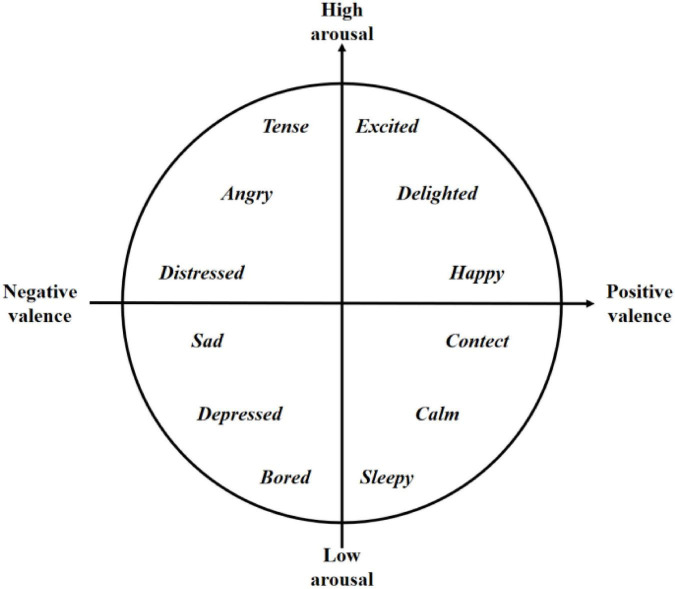
Valence-Arousal model of emotion.

Emotions themselves are highly complex and abstract, and psychologist Russell (1980) proposed a two-dimensional model of emotions, also known as the Valence-Arousal model (Figure 5), in which the horizontal and vertical axes represent Valence and Arousal, respectively. Since the two-dimensional model cannot effectively distinguish emotions such as fear and anger, Mehrabian proposed a three-dimensional spatial representation of emotions, adding “dominance” to Valence and Arousal, which is also known as the Valence-Arousal-Dominance model (Mehrabian, 1996).

Emotions play an extremely important role in the driver’s decision making, planning, reasoning and other behaviors (Ali et al., 2018). According to the Valence-Arousal model, the common driver emotions can be divided into positive emotions (e.g., happy, excited) and negative emotions (e.g., nervous, sad). Studies (Dula and Geller, 2003) have shown that emotions can significantly cause young drivers to engage in risky driving behaviors such as reckless driving and risky driving (Fernandes and Job, 2003), especially negative emotions. Positive emotions enable drivers to drive better, thus playing a certain positive role in traffic safety (Lewis et al., 2008). Steinhauser et al. (2018) studied the effects of positive and negative emotions on driving behavior and showed that emotions acted as a mediator to influence the driver’s attentional state and thus directly changed driving behavior. Existing research (Megías et al., 2011) demonstrates that drivers’ emotional states have a direct impact on their alertness, hazard awareness, and maneuverability (Pêcher et al., 2009). Drivers with irritability tend to drive fast and easily get irritated during driving, leading to aggressive driving behavior (Elander et al., 1993). For example, when their vehicle is forced to slow down due to the influence of other vehicles, they will have an angry mood, and then they will overtake the vehicle by repeatedly accelerating and changing lanes (Stephens and Groeger, 2009). Current research has focused on driving behavior in relation to risk perceptions, emotions, attitudes, and certain human characteristics, among which emotions and affective components highly influence human decision-making and perceived risk (Slovic et al., 2004). Barrett and Salovey (2002) suggested that emotions play a major role in motivational behavior. Analyzing the influence of the mood of driving behavior is also very meaningful, because it is difficult to change one’s character, but it can adjust and control the emotions, understand how the emotional states affect driving behaviors to affect driving behavior, is vital for development of advanced driver assistance system, the system through the flexibility to adapt to the current state of the driver to improve safety. This has great implications for reducing dangerous driving behavior.

At the cognitive level of the brain, the brain localization theory suggests that brain structure is closely related to emotions. Sarlo et al. (2005) used the film to induce negative emotion and neutral emotion of the subjects, respectively. The study found that the alpha band of EEG signals is highly related to emotional changes. When negative emotions are induced, the potentials of the alpha band in the right hindbrain will produce a strong response (Sarlo et al., 2005). The research from Balconi and Lucchiari (2008) and Miltner et al. (1999) indicated that two types of wave signals, gamma and beta, are particularly useful for recognizing emotion. Li and Lu (2009) observed that gamma wave signal was related with two particular but distinct emotions: happiness and sadness. On the other hand, Bos found that the most reliable electrode positions for detecting emotional valence are F3 and F (Lin et al., 2010).

Driver emotion recognition is becoming an important task for advanced driver assistance systems (ADAS), and data shows that monitoring the driver’s emotions while driving can provide important feedback to the driver, which can be useful in preventing accidents. Common emotion recognition methods can be divided into two categories based on non-physiological signals and physiological signals. Most of the initial studies used non-physiological signals such as facial expressions (Anderson and McOwan, 2006), voice intonation and physical features to extract recognition features (Yin et al., 2017), but the effect of recognition was not satisfactory because these features could be artificially and deliberately disguised. Physiological signal recognition mainly consists of two types, one is based on the peripheral nervous system, such as measurement of human heart rate, respiration, skin impedance, and physiological signals such as electromyography (Picard et al., 2001), and the other is based on the EEG signals of the central nervous system. The biggest advantage of peripheral nervous system-based recognition methods is that emotions are not easily artificial, but the disadvantage is the lack of a uniform criterion and low accuracy. With the continuous research, it has been found that emotions are closely related to human physiological and psychological activities, and the association with cortical activity is particularly obvious. The EEG signals also have the advantage of being less susceptible to artifacts because they contain a lot of physiological information, and they are more accurate than other physiological signals (Jie et al., 2014). It has become a hot and cutting-edge technology to study driver’s emotions through brain electricity. In 2016, a study (Zhang and Lee, 2010) made an effective distinction between positive and negative emotions in human beings through EEG. Zhang et al. (2011) classified the four emotions by an effective method combining GA-Fisher classifier and EEG, with an accuracy of 79.82–82.74%. Abhang et al. (2016) proposed an efficient and reliable emotion recognition system based on EEG signals.

Petrantonakis and Hadjileontiadis (2010) proposed a high-order crossover EEG feature of emotion recognition, and used this feature to identify emotion. Lin et al. (2010) induced 26 subjects into four emotions of joy, anger, joy and sadness, and then used linear classification algorithm to classify the collected EEG signals, with an accuracy of 79.23 and 85.35%. Nie et al. (2011) combined hybrid adaptive filtering with high-order crossover to classify six emotions, and the accuracy was 85.17%. Taking into account the driver’s personality characteristics and the influence of traffic environment, Fan et al. (2010) established a driver emotion detection model based on EEG signals using Bayesian network, which can provide adaptive assistance, and pointed out that in addition to alcohol and fatigue, emotion is another factor that affects driver behavior. Therefore, driver emotion detection can help improve driving safety. Chae (2015) found correlations between six emotions in simulated driving and brain signals evoked by flat-screen display images, suggesting that a novel driver vehicle interface could be designed. Rothkrantz et al. (2009) proposed a system of classification of certain emotional states by analysis of EEG signal, which can evaluate the drivers’ respondents under extreme emotions. Frasson et al. (2014) uses an EEG system to capture and analyses the type and intensity of the driver’s emotions, then generate corrective actions that can reduce the emotions. After a period of training, drivers are able to correct their emotions on their own (Frasson et al., 2014).

Katsis et al. (2008) proposed a method to evaluate the emotional state of a race car driver and designed a wearable system for emotion recognition, which assessed the emotional state by facial electromyography, ECG, respiration, and electrical skin activity, and validated the system. In future research on driving emotion recognition, extracting multimodal features will be beneficial to improve the accuracy of recognition models and provide strong support for the development of real-time driving emotion detection devices. Zheng et al. (2014) proposed an emotion recognition technique combining eye-movement information and EEG, whose experiment results show that the performance of the fusion model combining EEG and eye tracking features outperforms previous methods based on unimodal signals. Currently, multimodal-based driver state modulation is also a hot research topic to improve the overall model recognition accuracy.

## Discussion, Conclusion, and Future Prospects

It is necessary to study the EEG characteristics in different driving states, and to try to decipher the intrinsic correlation between driving behavior and brain activity from a neurological perspective, to study the intrinsic mechanism of driving behavior at a deeper level, and to apply it to future vehicle intelligent assistance systems, which can help improve the driving experience and enhance driving safety. This paper provides a review of the applications of EEG signals in the field of traffic safety, focusing on the monitoring of poor driving status, including fatigued driving, distracted driving, emotional driving, and some other applications. Given the above, understanding the states of drivers may lead to better state management.

In general, the advantages and limitations of applying EEG to driver state detection can be summarized as

### Advantages

Human cognitive states are closely related to human physiological and psychological activities, and the association with cortical activity is particularly obvious. Compared with other physiological signals, EEG signals can reflect the physiological activity of the human brain more intuitively and are more accurate since they are less susceptible to artifacts. Moreover, the EEG signals also have the advantages of high temporal resolution, non-invasiveness, low-cost properties.

### Limitations

The common head-mounted EEG measurement equipment used in EEG experiments are not very applicable in naturalistic driving conditions, because they are inconvenient to carry and the signals acquired by them fluctuate greatly when the drivers engage in various tasks (e.g., observing the surrounding traffic and reacting to a conflict) which can lead to uncontrolled interference to data collection. At present, the EEG experiments, which are non-invasive to the human brain and harmless to the psychology of the subjects, were usually conducted in driving simulators with a significant difference from real traffic environment. Unfortunately, there often is a certain arbitrariness in the behavior of the subjects since they think the driving errors will not lead to catastrophic consequences. Therefore, it is questionable whether the experiment results can truly reflect the EEG changes in real-life scenarios. Furthermore, the effectiveness of state recognition models based on EEG is closely related to study sample since the EEG signals have strong individual characteristics, which may cause the models trained in laboratory environment cannot deal with actual driving contexts very well.

Aiming at these limitations, some future research prospects were proposed to apply such technologies based on EEG to actual driving contexts and traffic safety improvement, as follow.

A portable acquisition device is the foundation for applying these state detection technologies based on EEG to the actual driving contexts. With the development of EEG dry sensors, low-power integrated circuits and wireless communication technologies, the EEG-based driver state detection under naturalistic driving conditions are considered to be promising. For instance, the drowsiness of professional drivers has been detected by simply wearing a cap with an EEG acquisition device in Australian coal mines.

A high-accurate and real-time state detection is necessary to deal with actual driving contexts. With the improvement of processing speed and computing power of computers, the machine learning approaches in a multi-modal setting, which can fully mine the complex data information and implicit features collected in unconstrained scenarios, are regarded as a viable bridge from research to practical use. For instance, a classification based on the dissimilarities of multi-modal physiological signals can efficiently recognize drivers’ emotions (Bota et al., 2019).

Automated driving is considered to be an effective means to avoid traffic accidents caused by poor driver driving status. Limited by technological development and legal establishment, the autonomous vehicles will be in human-machine co-driving phase for a long time. The current automated driving development does not consider the impact of bad driving states on driving behavior, and it is difficult to achieve accurate prediction of the driving behavior of drivers in different states. With the help of advanced machine learning algorithm techniques (Tan et al., 2022), the road traffic efficiency and safety will be significantly improved by applying the state detection technologies based on EEG to the practical use, such as an advanced brain-controlled driving assistance system or an automated driving system incorporating human brain cognitive decision-making and learning human driving behavior.

## Author Contributions

All authors contributed to the design and implementation of the review, to the analysis of the results and to the writing of the manuscript.

## Conflict of Interest

The authors declare that the research was conducted in the absence of any commercial or financial relationships that could be construed as a potential conflict of interest.

## Publisher’s Note

All claims expressed in this article are solely those of the authors and do not necessarily represent those of their affiliated organizations, or those of the publisher, the editors and the reviewers. Any product that may be evaluated in this article, or claim that may be made by its manufacturer, is not guaranteed or endorsed by the publisher.
